# Evaluating Simplified Web Interfaces of Risk Models for Clinical Use: Pilot Survey Study

**DOI:** 10.2196/22110

**Published:** 2021-07-16

**Authors:** Louis Beaubien, Colin Conrad, Janet Music, Sandra Toze

**Affiliations:** 1 Rowe School of Business Faculty of Management Dalhousie University Halifax, NS Canada; 2 School of Information Management Faculty of Management Dalhousie University Halifax, NS Canada

**Keywords:** risk model, electronic records, user interface, technology acceptance

## Abstract

**Background:**

In this pilot study, we investigated sociotechnical factors that affect intention to use a simplified web model to support clinical decision making.

**Objective:**

We investigated factors that are known to affect technology adoption using the unified theory of acceptance and use of technology (UTAUT2) model. The goal was to pilot and test a tool to better support complex clinical assessments.

**Methods:**

Based on the results of a previously published work, we developed a web-based mobile user interface, WebModel, to allow users to work with regression equations and their predictions to evaluate the impact of various characteristics or treatments on key outcomes (eg, survival time) for chronic obstructive pulmonary disease. The WebModel provides a way to combat information overload and more easily compare treatment options. It limits the number of web forms presented to a user to between 1 and 20, rather than the dozens of detailed calculations typically required. The WebModel uses responsive design and can be used on multiple devices. To test the WebModel, we designed a questionnaire to probe the efficacy of the WebModel and assess the usability and usefulness of the system. The study was live for one month, and participants had access to it over that time. The questionnaire was administered online, and data from 674 clinical users who had access to the WebModel were captured. SPSS and R were used for statistical analysis.

**Results:**

The regression model developed from UTAUT2 constructs was a fit. Specifically, five of the seven factors were significant positive coefficients in the regression: performance expectancy (*β*=.2730; *t*=7.994; *P*<.001), effort expectancy (*β*=.1473; *t*=3.870; *P*=.001), facilitating conditions (β=.1644; *t*=3.849; *P*<.001), hedonic motivation (β=.2321; *t*=3.991; *P*<.001), and habit (β=.2943; *t*=12.732). Social influence was not a significant factor, while price value had a significant negative influence on intention to use the WebModel.

**Conclusions:**

Our results indicate that multiple influences impact positive response to the system, many of which relate to the efficiency of the interface to provide clear information. Although we found that the price value was a negative factor, it is possible this was due to the removal of health workers from purchasing decisions. Given that this was a pilot test, and that the system was not used in a clinical setting, we could not examine factors related to actual workflow, patient safety, or social influence. This study shows that the concept of a simplified WebModel could be effective and efficient in reducing information overload in complex clinical decision making. We recommend further study to test this in a clinical setting and gather qualitative data from users regarding the value of the tool in practice.

## Introduction

### Background

Information overload negatively affects the decision effectiveness of clinical medical staff and ultimately impacts patient safety [1-3]. Clinical medical staff who are tasked with assessing patient outcomes are often required to use complex outcome and risk models in a spreadsheet format. In response to this challenge, we developed a mobile web model that simplifies the information presented to clinical medical staff and expedites the decision process. However, new electronic technologies often face barriers to adoption that inhibit their use in clinical settings [4,5].

In this pilot study, we investigate sociotechnical factors known to influence technology adoption through potential user feedback and assessment of a mobile web model designed for clinical decision support. The unified theory of acceptance and use of technology (UTAUT) is one of the most widely used models to predict voluntary user adoption and behavior toward a given information system. Employed in user-centered research in mobile web [6], consumer, and clinical health [7,8] contexts, the model is well-validated against four key constructs: (1) performance expectancy, (2) effort expectancy, (3) social influence, and (4) facilitating conditions [9]. In recent expansions of the model, three additional antecedents (hedonic motivation, price value, and habit) were added to UTAUT [6]. We investigate these factors predicted to influence technology adoption decisions by applying the UTAUT2 model in the context of an interface designed for clinical medical staff to assess options for treating chronic respiratory illness among the general population.

### Interface Development

A web-based mobile user interface (hereafter referred to as “the WebModel”) was developed to improve functionality and information consumption, following the common approach of using models to provide clinical insight and cost-effectiveness models based on large data sets in Microsoft Excel (Microsoft Corp). Cost-effectiveness models are commonly developed as Excel spreadsheets [10,11] and provide calculated forecasts of health outcomes and treatment costs based on a variety of possible inputs including health status, demographic characteristics, and cost factors. However, the development of these models is complex [12], and validation is an intense process [10]. The possibility of user error in the construction of the cost-effectiveness projections and the difficulty in interpreting information increases with the intensity and number of calculations that must be performed and the volume of information presented to the user. In this specific case, information overload was caused by the added presence of calculation details in the Excel spreadsheets and the production of results being spread across multiple spreadsheet tabs.

**Figure 1:**
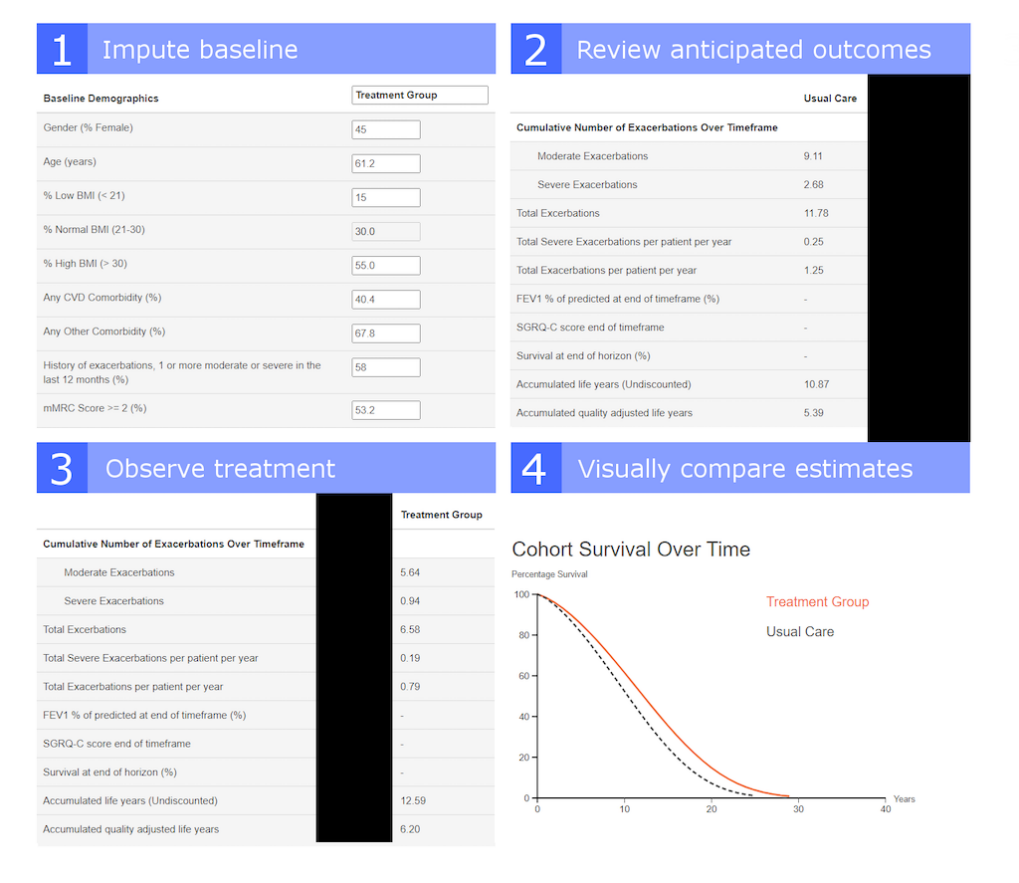
User flow of risk model calculator for respiratory disease interfaces.

The WebModel is designed to address issues of information overload through a simplified interface, to limit extraneous information (Figure 1). Information overload inhibits clinical medical staff from making effective decisions based on electronic medical records [13] and may contribute to errors or clinician burnout [14]. Other literature has found that overly informative medical data interfaces can increase clinical medical staff members’ cognitive workload, ultimately impairing patient outcomes [15]. Simplified electronic interfaces have been used to improve outcomes in intensive care units, which motivated our approach to design as we aimed to determine whether these results generalize to a less-urgent setting [16]. Although Excel provides a powerful and relatively simple way to perform complex calculations, including the large data sets and intricate calculations—in particular, nested regressions—often found in cost-effectiveness models taxes the capabilities of the software, compromises its reliability, and contributes to information overload [17]. We developed the WebModel using JavaScript to construct the mobile user interface and handle data interface between the presentation layer and the model. The underlying calculations are performed in equations modelled in Python on the server layer, which limits potential user errors and is a reliable means of performing complicated calculations [18]. The model is based on a data set of 20,000 simulated patient files. The data are intended to simulate response in the interface, rather than to represent clinical accuracy. The application at this stage is not meant to have clinical validity, but rather is being used to test the form and presentation of information in terms of its ability to visualize information. The next stage of investigation is to continue to develop the system and interface and introduce actual data.

The WebModel provides users the opportunity to work with the regression equations and their predictions to evaluate the impact of background characteristics or differences in treatment on long-term predictions of accrued survival time, exacerbation frequency, health-related quality of life (HRQL), and health resource use (HRU)/costs of treatment of chronic obstructive pulmonary disease (COPD) [10]. The WebModel shows the direct relation between an underlying model input (ie, specific characteristics related to a patient) and the linked outcomes. The goal is to limit the number of webform inputs presented to a user to between 1 and 20, depending on their desired comparison, rather than the dozens of detailed calculations that they would normally see in the Excel file. Figure 1 illustrates the elements of an output comparison of cohort survival of hypothetical treatment and nontreatment groups over time. The WebModel is intended to extend the understanding of respiratory illness through interactive access and provide clinical medical staff the opportunity to evaluate the model and the statistical relations it represents. The application uses a responsive design and can be rendered on either desktop or mobile devices by any user’s device that can access the website. It only takes information about cohorts and calculates results in the front end; in the current version, no patient information is transmitted by the user’s computer or back to the website origin server.

## Methods

A minimum viable mobile app was developed to replicate the use of a cost-effectiveness model. As noted above, simulated patient data populates the system, and an interface allows the clinical user to input patient demographic and statistical data (eg, age, height, weight). The system also allows the user to input patient information on the presentation of respiratory illness (eg, incidence of coughing spasms). The resulting outputs would indicate possible therapeutic approaches, and provide (simulated) historical outcomes. An adaptation of the UTAUT scale [6] is used to collect user responses on the perceived usefulness and usability of the system. The research is focused on the assessment of the usability of the interface and the value it may provide. The study did not examine the clinical value of the WebModel.

A questionnaire (Multimedia Appendix 1) was designed with items adapted from a prior literature review and administered online to 1231 clinical users with access to the WebModel interface. The study population was 843/1231 (68.5%) male, 4/1231 (0.32%) nonbinary, and the rest female. We targeted clinical medical staff who were within 5 years of completing their program, with 1114/1231 (90.5%) aged 20-29 years. As the questionnaire did not request it, we do not have demographic information for the respondents. The study was live for one month, and participants could start the study and continue it over the month. This design provided flexibility, in recognition of the busy schedules of the participants, and allowed them to play with the interface over time. We speculate, however, that this design might have led to a high dropout or incomplete rate because participants began the survey but forgot to come back to complete it. Due to incomplete surveys, we conducted analysis on 674 observable results. The questionnaire had two sections consisting of 29 items from the major constructs included in the proposed model. The first section probed respondents on the perceived efficacy of the WebModel. The second section included the original items from the UTAUT2 [9], modified and adapted from the mobile web framework to include the following: performance expectancy, effort expectancy, social influence, facilitating conditions, hedonic motivations, value, habit, and behavioral intent. Questions in the second section were measured using a 7-point Likert scale ranging from “strongly disagree” (1) to “strongly agree” (7).

Statistical analysis was conducted with IBM SPSS Statistics (version 25; IBM Corp) and the R programming language. As this is an exploratory study, we did not investigate actual use behavior and instead investigated the factors that influence behavioral intention to use. Reliability of the attitudinal scales was assessed by calculating internal consistency (Cronbach α) and multiple linear regression was used to explore the significance of the antecedents of adoption.

## Results

Multiple linear regression of the UTAUT2 factors to predict behavioral intention to use the WebModel was significant (*F*_7,672_=135.4; *R^2^*=0.5851; *P*<.001). We found that 5 of the 7 factors were significant positive coefficients in the regression: performance expectancy (*β*=.2730; *t*=7.994; *P*<.001), effort expectancy (*β*=.1473; *t*=3.870; *P*<.001), facilitating conditions (*β*=.1644; *t*=3.849; *P*=.001), hedonic motivation (*β*=.2321; *t*=3.991; *P*<.001), and habit (*β*=.2943; *t*=12.732; *P*<.001). Social influence was not a significant factor in the regression (*β*=–.0149; *t*=–.661; *P*=.51). Price value was found to have a significant negative influence on the regression (*β*=–.1566; *t*=–4.406; *P*<.001).

## Discussion

### Principal Findings

Ash found that more than 80% of health information technology adoption is not related to the underlying data in the system, but to the presentation of the information [19]. The results of the WebModel study suggest there are multiple influences on intent to use the application, many of which may relate to its technical efficiency in presenting clear information. However, the data suggest that sociotechnical factors [20] that include perceptions of how technology fits into workflow, interface design, and the perceived ability of the user also relate to the willingness to adopt the technology. One component that consistently impacts positive adoption is perceived failures in patient safety (eg, inaccurate output) resulting from system use [21,22]. As the WebModel was not tested for clinical use, and the system was deemed to be accurate and validated for the purpose of this study, it is not possible to draw conclusions about the impact of perceptions of patient safety. Future research would benefit from considering the WebModel as a valid model that might be applicable in clinical contexts. UTAUT constructs such as performance expectancy and effort expectancy were nonetheless positively correlated with intention to use the tool, perhaps reflecting that perceptions of time saved were determinants of use.

It was surprising that the price value of the system was found to be a negative factor in the decision to adopt the WebModel, as this is in contrast to the plurality of research on user adoption [6] that suggests perceptions of price value of the technology, or perceived value gained by using the technology relative to the price paid, will enhance adoption. One possible explanation is that health workers are removed from purchasing decisions in hospitals and are not equipped to weigh this factor. Alternatively, studies in extant literature suggest that an impediment to adoption of health technologies is the inability to input information in the form and expression that normal (nontechnological) workflows might allow [23]. For instance, while it may be possible to input values of moderate versus severe exacerbations in a model, there is no opportunity to make expressive additions to the notation. By contrast, entering information into a patient’s chart, or reading information from such a chart, allows for text written boldly, in all capitals, or circled aggressively, which might denote preference for more expressive options. Further study on user adoption of clinical decision support systems and cost-effectiveness models should examine perceptions of value gained through use of the system.

Finally, social influence was found to be nonsignificant in relation to adoption. User adoption literature suggests that peers would stimulate higher rates of adoption, and health care is no exception [24,25]. However, clinical users were provided access to the WebModel in isolation and provided no information that would suggest high or low rates of adoption or acceptance by peers. Therefore, the study is not adequately framed to examine the influence of peer users on the intention to adopt the technology. Future studies on system development and user adoption of the WebModel will examine the system operating in a clinical environment where user perceptions of value, usability, and utility of the system could be shared among study participants.

### Conclusion

Clinical decision making requires complex outcome and risk assessments based on large data sets, leading to information overload and the possibility of error. This pilot study investigated how a simplified mobile web model could reduce information overload in these situations and measured the usefulness and usability of this prototype. The results indicate that clinical medical staff would use the WebModel and find it useful, and that five of the key UTAUT2 factors significantly predicted intent. Further research to test the WebModel in a clinical setting is recommended to allow the factors of social influence and value to be examined as they relate to practice. The collection of qualitative data from users would help researchers better understand the value of this tool in relation to other systems, and how it might fit with workflows.

## Appendix

Multimedia Appendix 1 Questionnaire instrument.

## Abbreviations

COPD: chronic obstructive pulmonary disease
UTAUT: unified theory of acceptance and use of technology
