# Supplementary material for: Evaluating Simplified Web Interfaces of Risk Models for Clinical Use: Pilot Survey Study
Source: JMIR Form Res. 2021 Jul 16;5(7):e22110. doi: 10.2196/22110 (PMC8325085; doi:10.2196/22110)
Supplement: Multimedia Appendix 1 [file formative_v5i7e22110_app1.docx]

# MULTIMEDIA APPENDIX 1 – QUESTIONNAIRE INSTRUMENT

| **Item** | **Measure** | **Text** |
| --- | --- | --- |
| Q1 | Yes/No | Do you use one or more web application or model to do your job? |
| Q2 | Yes/No | Do you find the web model numerical display useful? |
| Q3 | Yes/No | Do you find the web model graphical display useful? |
| Q4 | Yes/No | Do you prefer numerical to graphical representation of data? |
| PE 1 | Likert (7) | I find the web model useful to my job. |
| PE 2 | Likert (7) | Using the web model helps me accomplish things more quickly. |
| PE 3 | Likert (7) | Using the web model increases my productivity |
| EE 1 | Likert (7) | Learning how to use the web model is easy for me. |
| EE 2 | Likert (7) | My interaction with the web model is clear and understandable. |
| EE 3 | Likert (7) | I find the web model easy to use. |
| EE 4 | Likert (7) | It is easy for me to become skillful at using the web model. |
| SI 1 | Likert (7) | People who are important to me think that I should use the web model. |
| SI 2 | Likert (7) | People who influence my behaviour think I should use the web model. |
| SI 3 | Likert (7) | People whose options that I value prefer that I use the web model. |
| FC 1 | Likert (7) | I have the resources necessary to use the web model. |
| FC 2 | Likert (7) | I have the knowledge necessary to use the web model. |
| FC 3 | Likert (7) | The web model is compatible with other technologies I use. |
| FC 4 | Likert (7) | I can get help from others when I have difficulties using the web model. |
| HM1 | Likert (7) | Using the web model is enjoyable. |
| HM 2 | Likert (7) | Using the web model is very entertaining. |
| V 1 | Likert (7) | Subscription web models are reasonably priced. |
| V 2 | Likert (7) | Medical web models are good value for the money. |
| V 3 | Likert (7) | Current prices for web models provide good value. |
| H 1 | Likert (7) | The use of the web model has become a habit for me. |
| H 2 | Likert (7) | I rely on using the web model. |
| H 3 | Likert (7) | I feel the need to use the web model. |
| BI 1 | Likert (7) | I intend to continue using the web model in the future. |
| BI 2 | Likert (7) | I will always try to use the web model in my workplace. |
| BI 3 | Likert (7) | I plan to continue to use the web model frequently. |
